# Global Epidemiologic Trends and Projections to 2030 in Non-Rheumatic Degenerative Mitral Valve Disease from 1990 to 2019: An Analysis of the Global Burden of Disease Study 2019

**DOI:** 10.31083/j.rcm2507269

**Published:** 2024-07-22

**Authors:** Chengmei Wang, Menglin Song, Hao Chen, Pan Liang, Gang Luo, Wei Ren, Sijin Yang

**Affiliations:** ^1^College of Integrative Medicine, Southwest Medical University, 646000 Luzhou, Sichuan, China; ^2^The Affiliated Traditional Chinese Medicine Hospital, Southwest Medical University, 646000 Luzhou, Sichuan, China

**Keywords:** non-rheumatic degenerative mitral valve disease, incidence, prevalence, deaths, DALYs, age-standardized rate

## Abstract

**Background:**

No studies have updated the epidemiologic changes in non-rheumatic degenerative mitral valve disease (DMVD) since 2019, thus this study utilized data from the Global Study of Diseases, Injuries, and Risk Factors 2019 (GBD2019) to assess the burden of DMVD in 204 countries and territories over the period 1990–2019, as well as changes in the prevalence, incidence, deaths and changes in disability-adjusted life years (DALYs).

**Methods:**

Using the results from the GBD2019, analyzing the incidence, prevalence, deaths, and DALYs rates, as well as their age-standardized rates (ASR). Based on the human development index (HDI), the socio-demographic index (SDI), age, and sex.

**Results:**

In 2019, there were 24.229 million (95% uncertainty interval (UI) 23.081–25.419 million) existing cases of DMVD worldwide, with 1.064 million (95% UI 1.010–1.122 million) new cases and 0.034 million (95% UI 0.028–0.043 million) deaths, and 0.883 million (95% UI 0.754–1.092 million) disability-adjusted life years. The incidence, prevalence, deaths, and DALYs of DMVD and their ASR showed significant differences across sex, age groups, regions, and countries from 1990 to 2019. It is projected that by 2030, the incidence of DMVD in females will be 0.72 million with an ASR of 15.59 per 100,000 population, 0.51 million in males with an ASR of 11.75 per 100,000 population, and a total incidence of 1.23 million with an ASR of 14.03 per 100,000 population.

**Conclusions:**

DMVD remains a significant public health problem that cannot be ignored, despite a decreasing trend in the ASR of global incidence, prevalence, deaths and DALYs from 1990 to 2019. However, we note an adverse development trend in countries with low socio-demographic indexes and seriously aging societies, and sex inequality is particularly prominent. This indicates the need to reposition current prevention and treatment strategies, with some national health administrations developing corresponding strategies for preventing an increase in DMVD based on local health, education, economic conditions, sex differences, and age differences.

## 1. Introduction

Degenerative mitral valve disease (DMVD) [1, 2] is characterized by degenerative changes in the connective tissue of the mitral valve with age, such as fibrotic degeneration and calcium deposition, resulting in mitral valve dysfunction causing hemodynamic changes. Mitral valve calcification, regurgitation, and stenosis can be secondary to abnormal heart load function, arrhythmias, heart failure, etc. Degenerative mitral valve disease lacks obvious clinical symptoms in theearly stages. Nevertheless, once combined with moderate-severe valve stenosis, incomplete closure, atrial fibrillation, or heart failure, it brings significant challenges for treatment, poor prognosis, and significantly shortened life expectancy [3, 4]. It has been calculated that the number of DMVD patients worldwide was approximately 24.23 million in 2019, and these prevalence numbers are expected to increase further. Moreover, the development of mitral valve disease is closely related to socioeconomic and cultural levels, with degenerative disease being the most common cause of DMVD in developed Western countries [5]. In contrast, mitral valve disease due to rheumatic pathology is more common in low-income countries [6]. With the rapid economic development of countries, the increasing aging of the population, and the rising incidence of chronic heart diseases, degenerative lesions will replace rheumatic lesions and gradually become the leading cause of mitral valve lesions [7]. For global health systems, this imposes an enormous public health burden and a noticeable increase in the consumption of social health resources and costs. Therefore, global DMVD prevention and control is quite critical. However, no studies have updated the epidemiological changes of DMVD since 2019, and the 2017 epidemiological studies did not deeply penetrate the effect of different regions, countries, age, and sexes [8].

This study relies on data from the Global Study of Diseases, Injuries, and Risk Factors 2019 (GBD2019) to provide a standardized and detailed analysis of the burden of DMVD [9]. Evaluating the trends of DMVD at the global, regional and national levels can help individual countries develop effective management strategies, rational allocation of medical resources to improve the outcomes of DMVD, and enhance patients’ quality of life.

## 2. Materials and Methods

### 2.1 Data Source

The GBD2019 study is an update and extension of GBD2017, a study that quantifies health losses from 204 countries and 22 regions for 369 diseases and 87 risk factors, using high-quality literature from various national health department administrative reports and researchers [9]. The Socio-Demographic Index (SDI) serves as a comprehensive measure for evaluating the level of development in each country or region. This index is calculated as the geometric mean of three components: the total fertility rate among female younger than 25 years old, the average educational attainment of females aged 15 and older, and the income per capita. These 204 countries are divided into five SDI regions depending on their SDI, which include low, low-middle, middle, high-middle and high SDI regions [9]. Theoretically, regions or countries possessing an SDI score of 0 are indicative of the minimal level of development concerning health-related indicators. In contrast, an SDI score of 1 in regions or countries theoretically denotes the apex of developmental achievement. In addition, these 204 countries and territories are grouped geographically within 21 GBD regions. The incidence, prevalence, deaths, disability-adjusted life years (DALYs), and their relative age-standardized rates (ASR) of DMVD in this study were gathered through the GBD Outcomes Tool (http://ghdx.healthdata.org/gbd-results-tool). The ASR was calculated using the GBD World Population Standard. All estimates were provided with 95% uncertainty intervals (UI).

### 2.2 DMVD Definition

DMVD is defined in standard terms according to the International Classification of Diseases (ICD). The codes linked to DMVD according to the ICD9 and ICD10 are 424.0 and I34.0, respectively.

### 2.3 Statistical Analyses

We quantified the global burden of DMVD by using the ASR of incidence, prevalence, deaths, and DALYs, as well as the estimated annual percentage change (EAPC) in prevalence [10]. When comparing the variability of rates with different regions, populations, and age structures, the use of crude rates is inaccurate, and to ensure the comparability of statistical indicators, different weights need to be given to crude rates according to age composition and age standardization is performed to obtain ASR. The unit of ASR is per 100,000 people and is calculated by the formula: $A\,S\,R=\frac{\sum_{i=1}^{A}a_{i}\,w_{i}}{\sum_{i=1}^{A}w_{i}}\times 100000$. In this context, “i” represents the specific age group in question, “$a_{i}$” signifies the rate of deaths specific to the age group “i”, and “$w_{i}$” indicates the proportional weight of age group “i” within the chosen reference standard population. ASR trends can serve as a great proxy for changes in the population’s disease modality and can guide changing risk factors. Assuming that the natural logarithm of ASR is linear over time, the annual trend in ASR indicates a stable change in the metric over time. Therefore, we use EAPC to measure the ASR trend over a specific time interval. Fitting a straight line gives Y=α+β⁢x+ε, Y: ln(ASR); x: year of calendar; ε: error. Similarly, EAPC has a 95% confidence interval (CI). The trend of ASR was considered to increase when both the estimate of EAPC and their 95% CI were greater than 0. Conversely, the ASR showed a decreasing trend when the EAPC estimate and its 95% CI were less than 0. Otherwise, ASR was considered stable over time [11]. At the national level, the associations between EAPC with the ASR (1990) and human development index (HDI) (2019) were assessed separately for the factors influencing EAPC. Correlations were used to assess the relationship between ASR with different SDI regions and HDI countries. Furthermore, to explore the temporal trends in the decomposition of changes in DALYs since DMVD is an age-related disease: the present study used the decomposition method established by Gupta [12, 13, 14]. The DALYs for DMVD were accounted for by four key elements: (1) the magnitude of the adult population, (2) the age composition of this population, (3) the prevalence of diseases associated with aging, and (4) the death rate and severity of these age-related conditions. The specific calculation formulas used were as follows:

$$D\,A\,L\,Y_{y}=\sum_{a,d}p\,o\,p\,s\,i\,z\,e_{y}.\frac{p\,o\,p\,a\,g\,e_{a\,y}}{p\,o\,p\,s\,i\,z\,e_{y}}.\frac{p\,r\,e\,v\,a\,l_{a\,d\,y}}{p\,o\,p\,a\,g\,e_{a\,y}}.\frac{D\,A\,L\,Y_{a\,d\,y}}{p\,r\,e\,v\,a\,l_{a\,d\,y}}$$,

y: year; a: age group; d: disease. To predict the burden of DMVD globally, this study used a Bayesian Age-Period-Cohort (BAPC) model to forecast the incidence of DMVD from 2020 to 2030 [15]. 

Statistical analyses and data visualizations were conducted utilizing R software (version 4.2.1, RStudio, AT&T Bell Laboratories, University of Auckland, New Zealand) and GraphPad Prism (version 9.5.1, Inc. San Diego, CA, USA). A *p*-value below 0.05 was deemed statistically significant.

## 3. Results

### 3.1 Global DMVD Disease Burden Trends 1990–2019

**Fig. 1.**
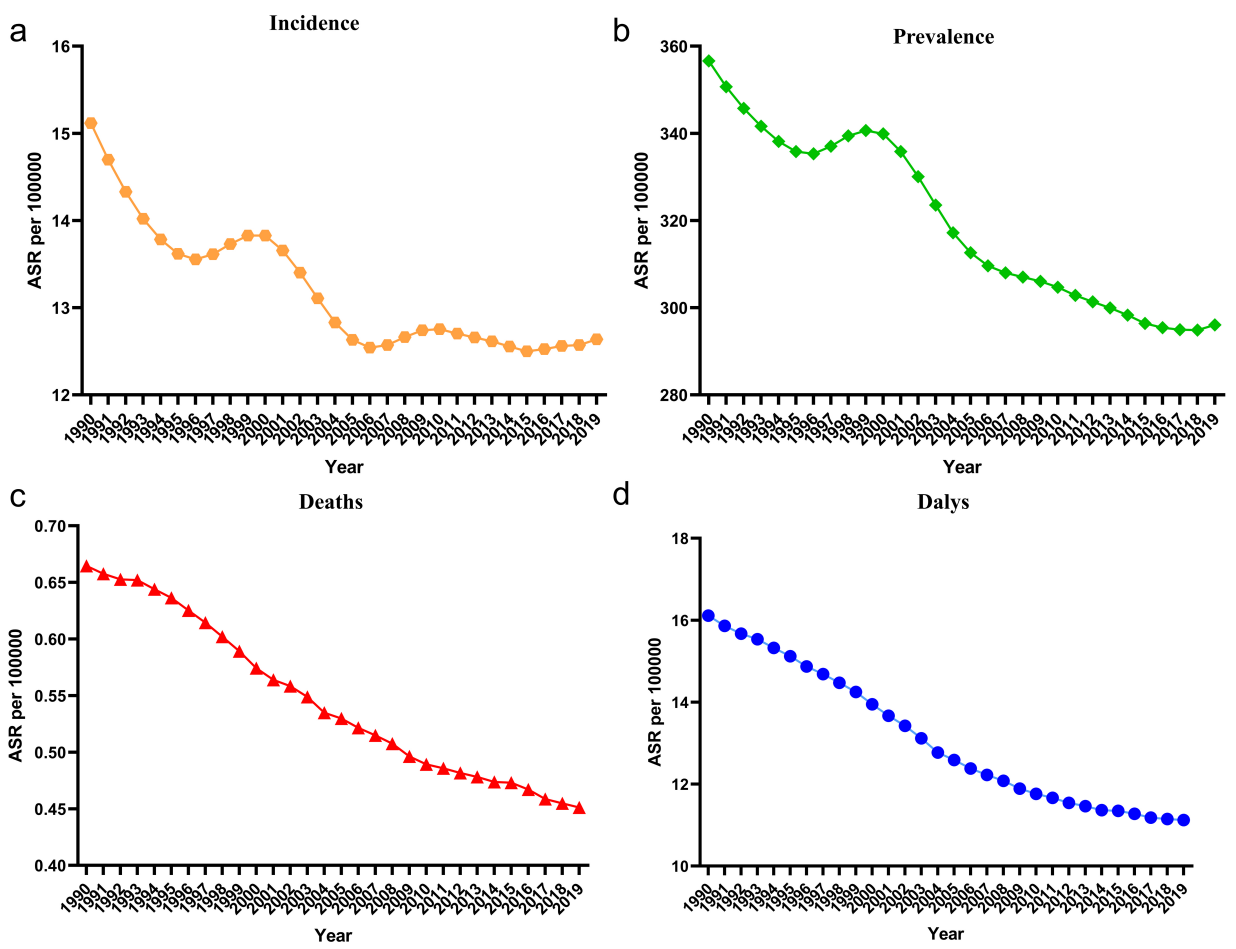
**The change trends in age-standardized incidence, prevalence, deaths and DALYs of patients with DMVD from 1990 to 2019.** (a) Incidence. (b) Prevalence. (c) Deaths. (d) DALYs. ASR, age-standardized rate; DALYs, disability-adjusted life years; DMVD, degenerative mitral valve disease.

**Fig. 2.**
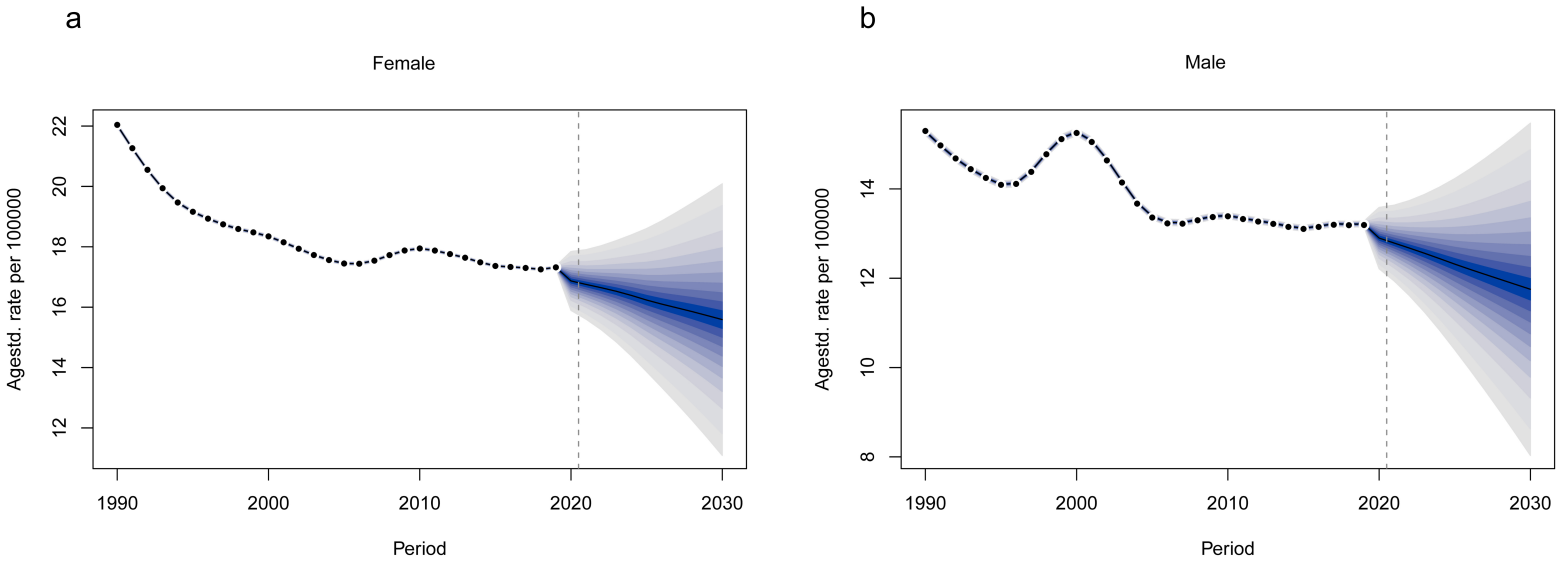
**BAPC model predicts age-standardized incidence of DMVD patients from 1990–2030.** (a) Female. (b) Male. BAPC, Bayesian Age-Period-Cohort.

The global burden of DMVD has increased significantly from 1990 to 2019 (Table 1). The incidences increased from 0.677 million (95% UI 0.644–0.714 million) in 1990 to 1.064 million (95% UI 1.010–1.122 million) in 2019, which represents a growth rate of 57.16%. The age-standardized incidence rate (ASIR) decreased from 15.117 per 100,000 (14.31–15.96) to 12.64 per 100,000 (12.00–13.32). The prevalence increased from 14.218 million (95% UI 13.503–14.950 million) in 1990 to 24.229 million (95% UI 23.081–25.419 million) in 2019, representing a growth rate of 70.41%. The age-standardized prevalence rates (ASPR) decreased from 356.64 per 100,000 (339.20–375.01) to 296.06 per 100,000 (282.38–310.48). The deaths increased from 0.022 million (95% UI 0.019–0.026 million) in 1990 to 0.034 million (95% UI 0.028–0.043 million) in 2019, which represents an increase of 54.55%. Age-standardized deaths decreased from 0.66 per 100,000 (0.53–0.78) to 0.45 per 100,000 (0.37–0.58). The DALYs increased from 0.626 million (95% UI 0.539–0.751 million) in 1990 to 0.883 million (95% UI 0.754–1.092 million) in 2019, representing a growth rate of 41.05%. The age-standardized DALYs rates decreased from 16.12 per 100,000 (13.81–19.28) to 11.12 per 100,000 (9.49–13.75). During the period 1990–2019, it found an interesting phenomenon, the age-standardized deaths and DALYs rates have been showing a gradually decreasing trend, while the incidence and prevalence all showed a decreasing trend until 1996 and a small peak in 1996–2000, after which the trend showed a gradual decrease and leveled off (Fig. 1). Fig. 2 and **Supplementary Table 1** show that the BAPC model predicts that the total incidence of DMVD in females is 0.72 million with an ASIR of 15.59 per 100,000 in 2030, and in males, 0.51 million with an ASIR of 11.75 per 100,000 and a total incidence of 1.23 million with an ASIR of 14.03 per 100,000.

**Table 1.** **The prevalence cases and ASPR of DMVD in 1990 and 2019, and its temporal trends from 1990 to 2019**.

Characteristics	1990		2019		1990–2019
	Prevalence cases	ASR per 100,000	Prevalence cases	ASR per 100,000	EAPC
	No. × ${\mathrm{10}}^{5}$ (95% UI)	No. (95% UI)	No. × ${\mathrm{10}}^{5}$ (95% UI)	No. (95% UI)	No. (95% CI)
Global	142.2 (135.0–149.5)	356.6 (339.2–375.1)	242.3 (230.8–254.2)	296.1 (282.4–310.5)	–0.68 (–0.74–0.62)
High SDI	82.8 (79.1–86.5)	824.5 (787.9–862.6)	131.5 (125.8–137.6)	785.6 (752.1–821.2)	–0.29 (–0.43–0.14)
High-middle SDI	47.9 (45.3–50.9)	443.7 (420.0–471.0)	81.2 (77.3–85.7)	400.2 (381.1–422)	–0.37 (–0.54–0.21)
Middle SDI	8.9 (8.2–9.7)	82.9 (76.4–89.8)	23.2 (21.7–25.0)	91.3 (85.4–97.9)	0.42 (0.35–0.49)
Low-middle SDI	2.2 (1.9–2.4)	33.9 (30.4–38)	5.4 (4.9–6.0)	37.9 (34.5–41.8)	0.43 (0.37–0.48)
Low SDI	0.39 (0.34–0.45)	14.1 (12.2–16.3)	0.92 (0.80–1.06)	14.8 (12.8–17.1)	0.15 (0.08–0.23)
Andean Latin America	0.027 (0.021–0.035)	11.5 (9–14.4)	0.071 (0.055–0.089)	11.9 (9.3–15)	0.09 (–0.01–0.19)
Australasia	0.57 (0.51–0.64)	241.6 (215.2–272.2)	1.1 (0.93–1.2)	225.8 (199.1–254.8)	–0.3 (–0.35–0.25)
Caribbean	0.052 (0.042–0.063)	18.6 (15.2–22.6)	0.098 (0.80–1.21)	19.1 (15.5–23.5)	0.09 (0.02–0.15)
Central Asia	2.0 (1.7–2.5)	417.7 (341.3–514)	4.0 (3.3–4.9)	507.4 (420.8–613.4)	1.0 (0.73–1.27)
Central Europe	9.2 (8.1–10.6)	624.3 (552.7–722.1)	14.2 (12.7–16.3)	713.9 (635.1–820.3)	0.68 (0.59–0.76)
Central Latin America	0.14 (0.12–0.16)	14 (12.2–16)	0.36 (0.32–0.42)	14.8 (12.8–16.9)	0.22 (0.09–0.34)
Central Sub-Saharan Africa	0.042 (0.033–0.052)	15.5 (12.4–19.3)	0.092 (0.071–0.12)	14 (11.1–17.5)	–0.39 (–0.54–0.23)
East Asia	15.7 (14.8–16.6)	174.2 (164.3–184.7)	48.2 (45.9–50.4)	230.1 (219.5–240.7)	1.1 (0.98–1.22)
Eastern Europe	12.6 (11.8–13.3)	460.2 (433.1–486.9)	15.4 (14.5–16.2)	478.2 (450.5–504.4)	0.33 (0.17–0.5)
Eastern Sub-Saharan Africa	0.11 (0.096–0.13)	12.8 (10.8–15.1)	0.25 (0.21–0.30)	12.2 (10.3–14.4)	–0.31 (–0.41–0.21)
High-income Asia Pacific	26.5 (25.3–27.7)	1297.4 (1238.8–1356.5)	43.4 (41.3–45.7)	1184.4 (1121.8–1246.7)	–0.49 (–0.56–0.42)
High-income North America	46.4 (44.6–48.5)	1370.5 (1314.8–1432.2)	67.8 (65.4–7.05)	1224 (1180.3–1270.3)	–0.57 (–0.84–0.3)
North Africa and Middle East	1.08 (0.89–1.29)	54.7 (45.3–65.7)	2.9 (2.4–3.5)	58.2 (48.5–69.4)	0.96 (0.62–1.3)
Oceania	0.021 (0.017–0.026)	62.2 (49.8–75.9)	0.049 (0.039–0.061)	61.3 (49–74.7)	–0.15 (–0.19–0.12)
South Asia	0.74 (0.66–0.82)	11.3 (10.2–12.4)	2.1 (1.9–2.3)	13.9 (12.6–15.3)	0.73 (0.68–0.79)
Southeast Asia	1.3 (1.1–1.5)	49.7 (43.3–56.9)	3.6 (3.1–4.2)	57.8 (50.1–66.5)	0.47 (0.43–0.51)
Southern Latin America	0.43 (0.35–0.53)	93.2 (76.3–115)	0.84 (0.68–1.04)	103.7 (84.4–128)	0.33 (0.3–0.36)
Southern Sub-Saharan Africa	0.055 (0.050–0.061)	17 (15.5–18.6)	0.10 (0.093–0.11)	15.7 (14.3–17.2)	–0.47 (–0.64–0.3)
Tropical Latin America	0.25 (0.23–0.26)	22.4 (20.9–23.9)	0.55 (0.51–0.59)	22 (20.5–23.5)	–0.11 (–0.18–0.05)
Western Europe	24.8 (23.7–26.2)	447.8 (427–471.1)	36.8 (35.3–38.5)	456.4 (437.5–477.1)	–0.15 (–0.33–0.03)
Western Sub-Saharan Africa	0.14 (0.12–0.16)	12.8 (11.1–14.6)	0.34 (0.29–0.39)	13.6 (11.8–15.5)	0.27 (0.21–0.34)

ASR, age-standardized rate; CI, confidence interval; EAPC, estimated annual percentage change; UI, uncertainty interval; DMVD, Non-rheumatic degenerative mitral valve disease; ASPR, age-standardized prevalence rates; SDI, socio-demographic index.

### 3.2 Global Age Distribution of DMVD Disease Burden in 2019

**Fig. 3.**
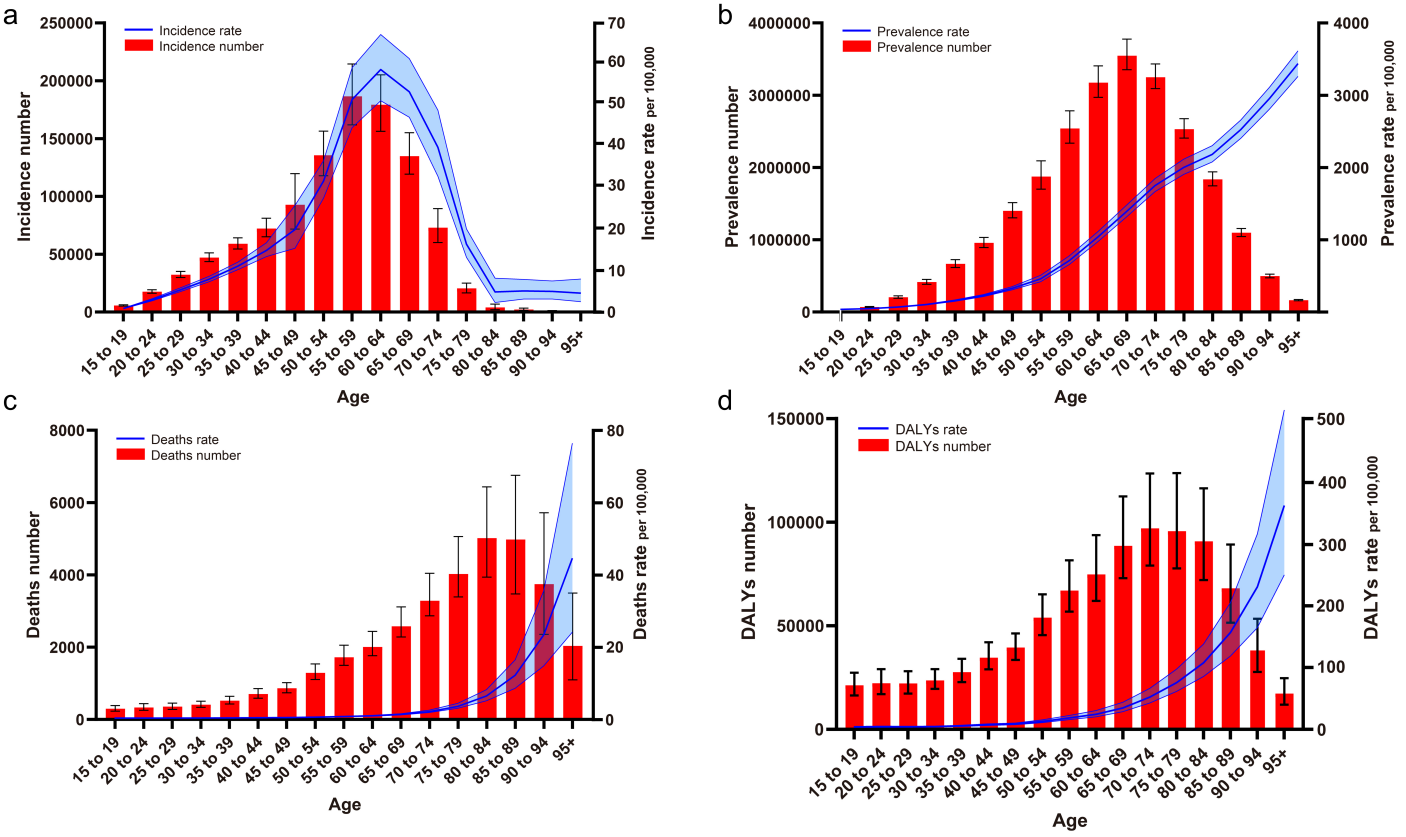
**Global changes in the epidemiology of DMVD patients in different age groups. **(a) Incidence. (b) Prevalence. (c) Deaths. (d) DALYs. DALYs, disability-adjusted life years; DMVD, degenerative mitral valve disease.

The global DMVD prevalence, deaths, and DALYs increase with age (**Supplementary Table 2**). In contrast, the incidence increases rapidly with age until age 65 years and peaks at age 60–64 years, declines rapidly after age 65 years, and plateaus after age 85 years. Fig. 3 illustrates these trends, the incidences gradually increases with age until age 60, peaks between 55–59 years, and then decline (Fig. 3a); the prevalence gradually increases with age until age 70, peaks between 65–69 years, and then decreases (Fig. 3b); the deaths gradually increases by age until the age of 85, peaks between 80–84 years, and then declines (Fig. 3c); and the DALYS gradually increases with age until age 75, peaks between 70–74 years, and then declines (Fig. 3d).

### 3.3 Sex Distribution of Global DMVD Disease Burden

**Fig. 4.**
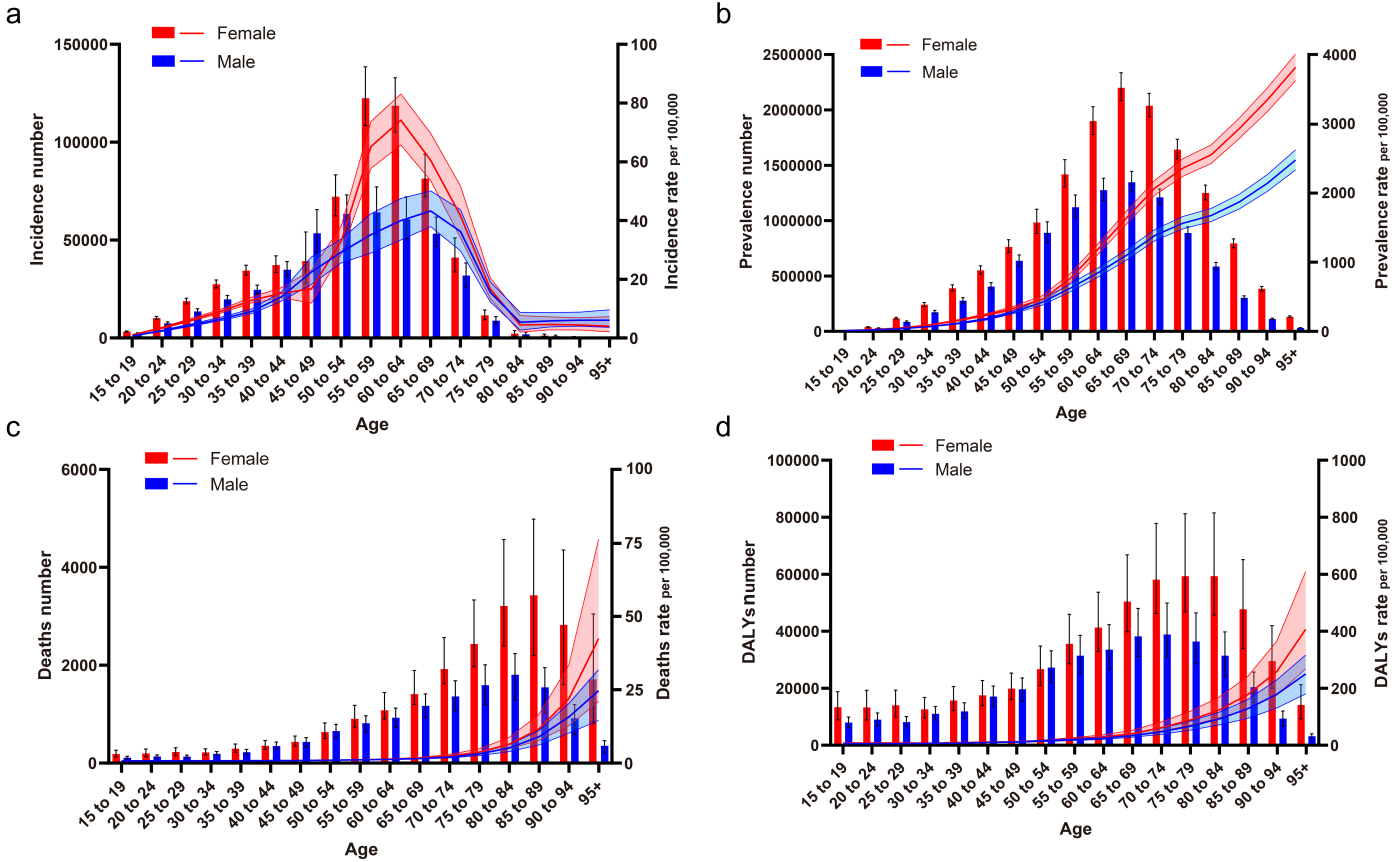
**Epidemiological changes of DMVD patients by sex at different ages. **(a) Incidence. (b) Prevalence. (c) Deaths. (d) DALYs. DALYs, disability-adjusted life years; DMVD, degenerative mitral valve disease.

The incidence, prevalence, deaths and DALYs, and their rates are slowly increasing for both males and females from 1990–2019, with an overall higher disease burden for females than for males when looking at overall numbers and trends (**Supplementary Fig. 1**). And from 2019, there were significant differences in the burden of disease between males and females at all ages, with females having a more substantial number of incidences than males overall (except for the 45–49 age group), especially after 50 years of age; in prevalence, all ages were greater than males, and in deaths, females had a higher number of deaths in all age groups than males (except for the 50–54 age group), in DALYs, females were consistently higher than males (except for the 50–54 age group) (Fig. 4). **Supplementary Tables 3,4** shows that the DMVD incidence rate in 2019 was 16.13/100,000 (15.31–17.02) for females and 11.38/100,000 (10.78–12.05) for males, the prevalence rate was 385.09/100,000 (367.06–403.14) for females and 241.64/100,000 (229.13–255.00), the deaths rate was 0.56/100,000 (0.44–0.76) for females and 0.33/100,000 (0.26–0.39) for males, and the DALYs rate was 13.70/100,000 (11.37–17.81) for females and 9.14/100,000 (7.52–10.99) for males. As seen in **Supplementary Fig. 2**, female incidence and prevalence are higher than male across SDI regions, and both indicators are positively correlated with SDI levels (**Supplementary Fig. 2a,b**). In addition, deaths and DALYs are also higher for females than for males, but these two indicators show a U-shaped distribution across SDI levels (**Supplementary Fig. 2c,d**).

### 3.4 Global Distribution of DMVD Disease Burden among Regions and Countries

**Fig. 5.**
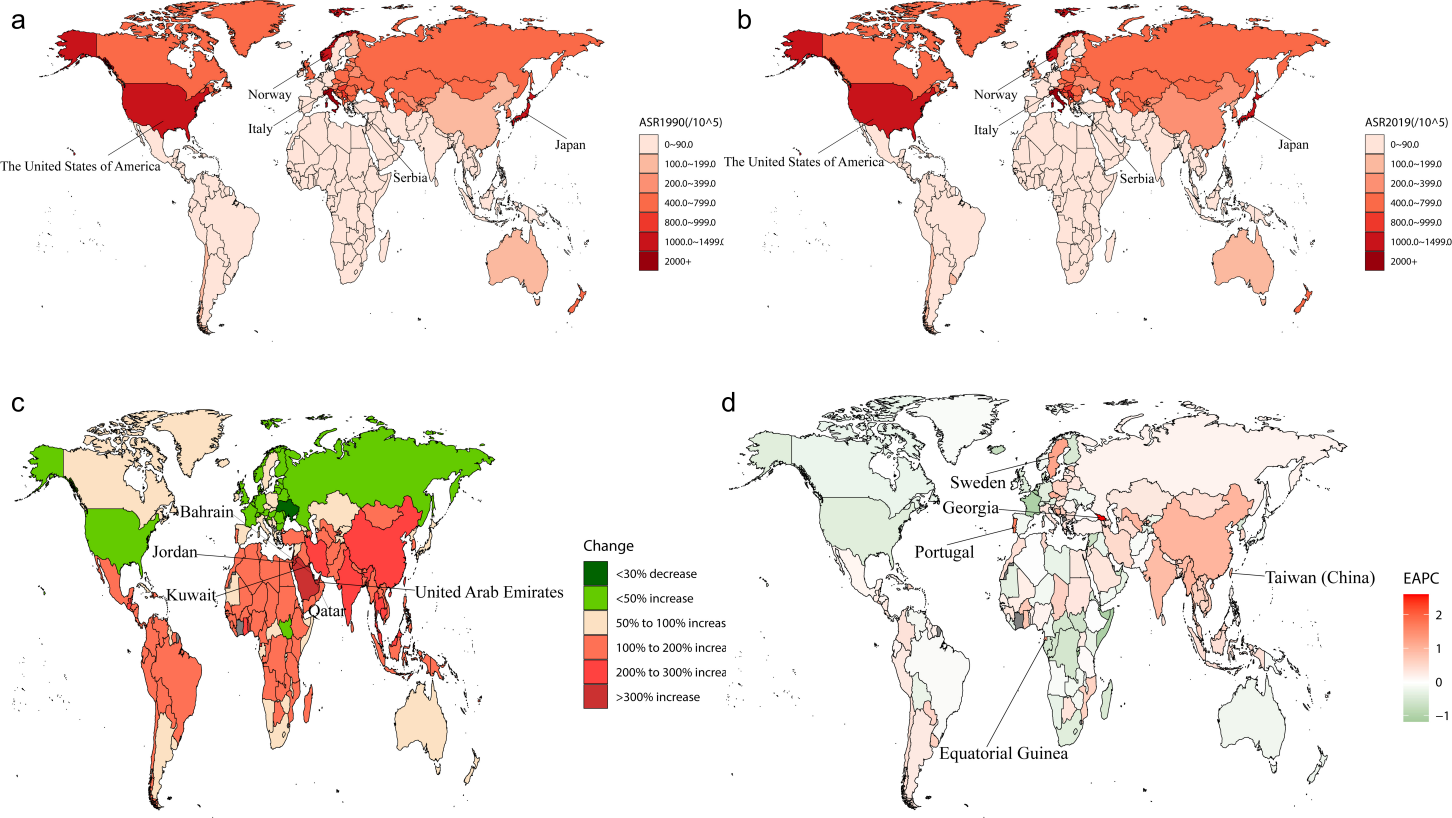
**Geographic heat map.** (a) ASR of DMVD patient prevalence in 1990. (b) ASR of DMVD patient prevalence in 2019. (c) Change in prevalence 1990–2019. (d) EAPC of prevalence 1990–2019. ASR, age-standardized rate; EAPC, estimated annual percentage change; DMVD, degenerative mitral valve disease.

The geographic distribution heat map (Fig. 5) indicates that in 1990 the DMVD burden varied considerably between countries. Upon adjusting for age, the leading five countries were identified as Italy (2185.9 per 100,000), Norway (1498.0 per 100,000), Japan (1467.5 per 100,000), the USA (1459.6 per 100,000), and Serbia (877.5 per 100,000). In 2019, after standardization for age, the top five nations were Italy (2384.4 per 100,000), Japan (1436.0 per 100,000), Norway (1433.6 per 100,000), the USA (1311.2 per 100,000), and Serbia (1043.9 per 100,000). From 1990 to 2019, the five leading countries and regions globally in terms of the rise in prevalence rate were Qatar (778.0%), United Arab Emirates (708.8%), Bahrain (443.8%), Jordan (426.6%) and Kuwait (348.9%). After standardization for age, the top five nations in terms of EAPC were Georgia (2.60), Portugal (1.67), Equatorial Guinea (1.30), Sweden (1.19), and Taiwan (China) (1.05).

### 3.5 DMVD Disease Burden on the Level of Socioeconomic Development

#### 3.5.1 Trends in DMVD Disease Burden at Different SDI Levels, 1990–2019

**Fig. 6.**
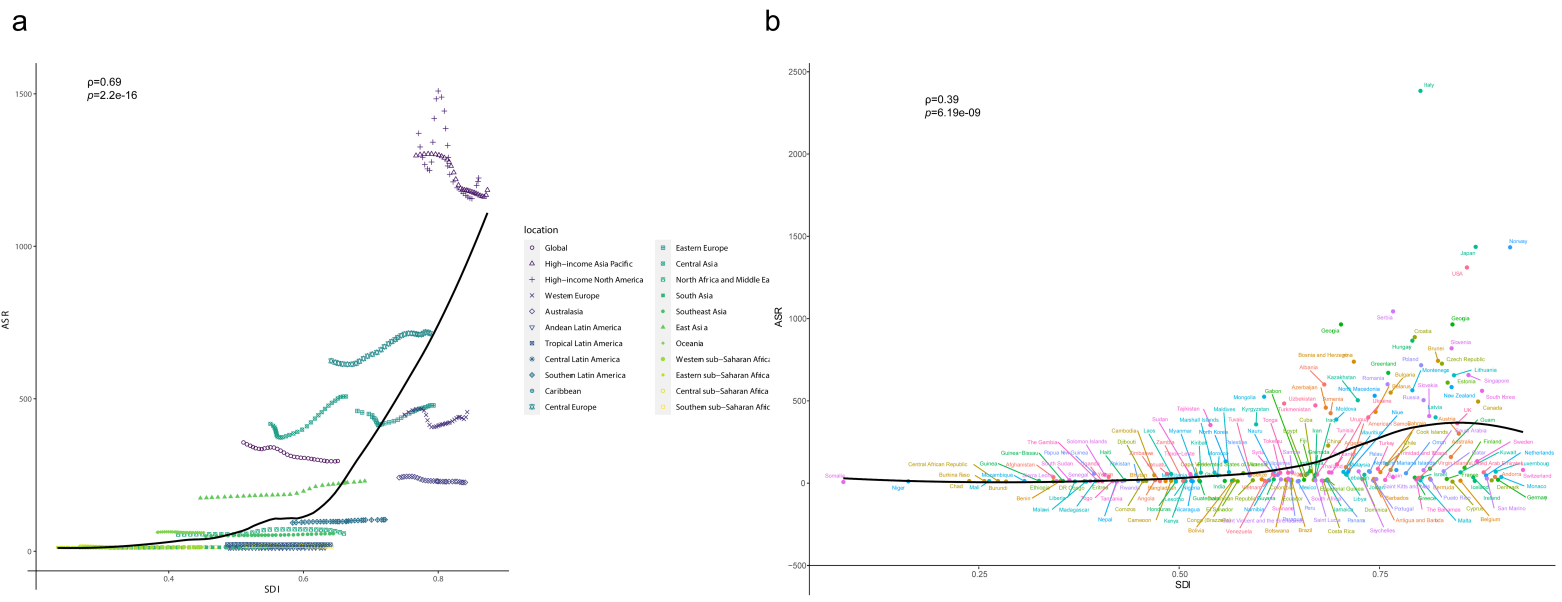
**Rates of DMVD age-standardized DALYs by SDI for 21 GBD regions (a) and 204 countries (b), 1990–2019, with expected values based on SDI and disease rates for all localities shown as black lines. **GBD, Global Burden of Disease, Injury, and Risk Factor Study; ASR, age-standardized rate; SDI, socio-demographic index; DMVD, degenerative mitral valve disease; DALYs, disability-adjusted life years.

This study showed significant differences in disease burden changes among the five SDI regions. **Supplementary Fig. 3** illustrates that the ASR for incidence, prevalence, deaths, and DALYs were markedly higher in regions with middle-high and high SDI compared to those with middle, middle-low, and low SDI. However, there was a rapid downward trend observed in the ASR of death and DALYs in these areas. As shown in Fig. 6a, the age-standardized prevalence of DMVD tended to increase quickly from low to high SDI regions, especially in areas with SDI >0.6, ρ = 0.69, *p* = 2.2 × ${\mathrm{10}}^{-16}$. Fig. 6b shows a gradual increase in age-standardized prevalence with increasing SDI in 204 countries in 2019, with Italy, Japan, the United States, and Norway showing exceptionally high standardized prevalence, ρ = 0.39, *p* = 6.19 × ${\mathrm{10}}^{-9}$.

#### 3.5.2 EAPC with ASR/HDI Relationship

**Fig. 7.**
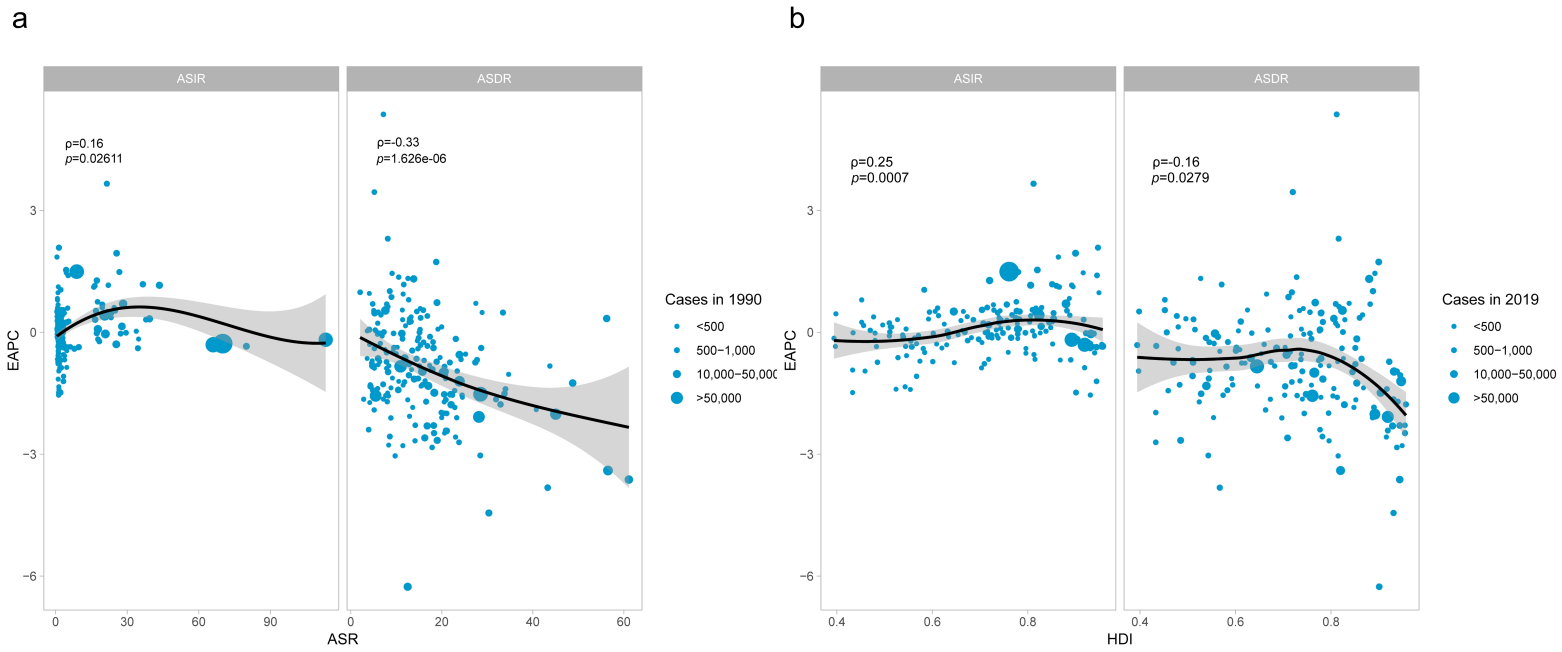
**The correlation between EAPC and DMVD ASR in 1990 (a) and HDI in 2019 (b).** The circles represent countries that were available on HDI data. The size of circle is increased with the cases of DMVD. The ρ indices and *p*-values were derived from Pearson correlation analysis. EAPC, estimated annual percentage change; ASR, age-standardized rate; HDI, human development index; DMVD, degenerative mitral valve disease; ASIR, agestandardized incidence rate; ASDR, agestandardized DALYs rate; DALYs, disability-adjusted life years.

There was a strong association between EAPC with ASR (1990) and HDI (2019), respectively, as shown in Fig. 7. The ASR for DMVD in 1990 serves as a baseline representation of the disease, while the Human Development Index (HDI) in 2019 acts as a proxy for the level and accessibility of medical care in each country. There was a remarkable positive association between EAPC and ASIR, with ASIR being restricted to less than 45/100,000 (ρ = 0.16, *p* = 0.026). In contrast to the ASIR trend, age-standardized DALYs rates (ASDR) was consistently and significantly negatively correlated with EAPC (ρ = –0.33, *p* = 1.63 × ${\mathrm{10}}^{-6}$) (Fig. 7a). From the HDI perspective, the level of EAPC in ASIR increased with a gradual increase in HDI (ρ = 0.25, *p* = 0.00071). And the level of EAPC in ASDR was significantly lower in high HDI regions than in middle and low HDI countries (ρ = –0.16, *p* = 0.0028) (Fig. 7b).

#### 3.5.3 Decomposition Analysis of DALYs

The results of the study showed that global population growth during 1990–2019 resulted in an increase in the burden of DALYs by 140.15%, population aging (73.13%), disease prevalence (–58.91%), and disease severity (–56.35%). The contribution of population aging to DALYs was most pronounced in the high and high-middle SDI (193.09%, 120.11%) quintiles, followed by a decreasing trend in the middle, low-middle and low SDI quintiles (58.54%, 23.30% and –2.46%, respectively). The epidemiology showed different trends globally, with positive epidemiological contributions from middle, low-middle and low SDI regions (7.59%, 13.44% and 10.49%, respectively) and negative contributions from high and high-middle SDI regions (–9.64% and –36.08%, respectively). There was an overall increasing trend in disease severity from high SDI to low SDI areas (–234.16%, –113.13%, –73.94%, –32.79%, and –48.17%, respectively). However, no significant difference was observed in population growth (150.70%, 129.11%, 107.81%, 96.06%, and 140.13%, respectively) (Table 2).

**Table 2.** **Changes in DALYs by population-level determinants from 1990 to 2019 globally and by Sociodemographic Index quintile**.

Location	Overall difference	Change due to population-level determinants (contribution to the total change)			
		Population	Aging	Epidemiological change	Severity
Global	257,546.8306	360,939.26 (140.15%)	188,333.20 (73.13%)	–146,587.57 (–56.92%)	–145,138.0 (–56.35%)
High SDI	49,772.20508	75,005.56 (150.70%)	96,107.47 (193.10%)	–4795.64 (–9.63%)	–116,545.0 (–234.16%)
High-middle SDI	49,036.49256	63,310.12 (129.11%)	58,896.630 (120.11%)	–17,693.67 (–36.08%)	–55,476.6 (–113.13%)
Middle SDI	54,870.8643	59,155.34 (107.81%)	32,123.40 (58.54%)	4164.63 (7.59%)	–40,572.5 (–73.94%)
Low-middle SDI	65,458.6466	62,876.59 (96.06%)	15,252.10 (23.30%)	8796.70 (13.44%)	–21,466.7 (–32.79%)
Low SDI	38,242.04388	53,589.44 (140.13%)	–938.92 (–2.46%)	4012.19 (10.49%)	–18,420.7 (–48.17%)

SDI, socio-demographic index; DALYs, disability-adjusted life years.

## 4. Discussion

This study provides a detailed analysis of four epidemiological indicators of global DMVD, namely incidence, prevalence, deaths, and DALYs from 1990–2019, based on GBD 2019 data. The study shows that the burden of DMVD is gradually increasing globally. Over the past 30 years, the incidences increased by 57.16%, the prevalence increased by 70.41%, the deaths increased by 54.55%, and the DALYs increased by 41.05%. In 2019, the number of new cases of DMVD was 1.064 million, the prevalence was about 24.229 million, the deaths were about 0.034 million, and the DALYs were about 0.883 million. It is comforting to note that the ASRs for all outcomes are exhibiting a decline, indicating a reduction in the real incidence of DMVD when adjusted for age demographics. With the aging of the global population, there is an increase in the absolute cases of DMVD. However, this increase may not be apparent in the ASR, possibly due to shifts in demographic patterns, enhancements in healthcare, and heightened awareness of health among the population. Based on the BAPC model predictions, the total incidence of DMVD during 2030 in females will be 0.72 million with an ASIR of 15.59 per 100,000, and in males, 0.51 million with an ASIR of 11.75 per 100,000 and a total incidence of 1.23 million with an ASIR of 14.03 per 100,000. The growing burden on public health necessitates considering factors such as population dynamics, the quality of health services, the efficacy of preventive measures, and socio-economic influences when analyzing disease burden and devising public health strategies.

Observing global patterns in DMVD epidemiology reveals that the prevalence rate of DMVD is progressively decreasing in the majority of high-income countries, serving as a model for other nations. However, it is concerning that the prevalence of DMVD is rapidly rising across Asia. This trend could be associated with the region’s swift economic growth in recent years, the significant aging of the population in most Asian countries, and the shift in the cause of valve disease from rheumatic factors to degenerative diseases among the elderly [8, 16]. In addition, due to the gradual westernization of the Asian diet and the increasing incidence of coronary heart disease [17], non-rheumatic ischemic mitral regurgitation disease will gradually increase with the increase of coronary heart disease [18]. It will be diagnosed as DMVD on ultrasound, leading to a gradual increase in the prevalence of DMVD. When pinpointed to countries, both in terms of the number of prevalence and ASPR, countries with a greater burden of DMVD have significant aging populations, most notably Japan, Italy, and the United States, with high numbers of prevalence and ASPR. The study also found an interesting phenomenon that countries in the Middle East have incredibly high growth rates in terms of prevalence. Unlike other countries with aging populations, aging in the Middle East is not severe [19, 20]. The dramatic increase in prevalence in the Middle East can be attributed to the high economic development in recent years, which has led to a significant increase in the availability of medical resources and a significant increase in the detection of DMVD. In addition, the population in the Middle East is changing its diet to one high in fat, sugar, salt and cholesterol, and the prevalence of hypertension and diabetes is gradually increasing [21]. At the same time, degenerative mitral valve disease is closely related to diabetes, hypertension, and coronary heart disease [18], all of which contribute to the rapid increase in the prevalence of DMVD in the Middle East. We can therefore target increased healthcare spending on diseases such as coronary heart disease, diabetes, and hypertension, combined with some of the more advanced public health approaches in terms of prevention and treatment policies in European countries.

There are also significant sex and age differences in the disease burden of DMVD. The rate of prevalence, deaths, and DLAYs of DMVD gradually increased with increasing age. However, the incidence decreases progressively after 65 years, which has not been observed in the past. This could be because co-morbidities with common risk factors for DMVD lead patients to seek medical care before age 65, the incidence of which gradually decreases after age 65. The severe burden of DMVD is concentrated in groups after age 55, and aging is a crucial factor in constituting DMVD. With aging, the tissues of the mitral valve gradually harden and lose their elasticity, becoming susceptible to long-term cardiac stress, leading to the development of DMVD. Studies show that the declining metabolic rate in the elderly may lead to glucose and lipid metabolic disorders, which may increase the risk of mitral valve disease, and hypertension is a significant risk factor for DMVD [22]. As population aging progresses, the number of people with hypertension will also increase, thus increasing the risk of developing DMVD [23]. The overall burden is significantly higher for females compared to males at almost all ages in 2019, with this difference being particularly pronounced in the postmenopausal period. Moreover, in various SDI regions, females also bear a higher burden. Some studies suggest that estrogen changes may lead to changes in the cardiovascular system, including decreased protection of vascular endothelial cells, decreased oxidoreductase activity, and increased inflammation [24], which may lead to thinning and stiffening of the mitral valve, thereby increasing the risk of DMVD in females [25]. Studies have found that the physiology of the heart is different in females and males. The mitral valve in females is usually smaller than in males and is more susceptible to stress, resulting in degenerative changes such as calcification, stiffening, and loss of elasticity [26]. In addition, researchers have found that episodes of autoimmune disease are associated with mitral valve disease and that females are more likely than males to develop autoimmune diseases such as systemic lupus erythematosus, which can cause heart valve damage [27, 28]. However, these burdens are reflected in the number of diseases and the considerable sex inequalities in surgical treatment. The males dominate surgical treatment [29]. Notably, the threshold values indicating the size of the heart requiring surgery were established primarily using the male population [30]. At the same time, fewer females met the recommended surgical criteria for ventricular enlargement in mitral valve disease. Furthermore, economic circumstances partially underpin this issue, as the diminished quality of life and lowered productivity in females with DMVD may exacerbate the income disparity between males and females. This widening gap could lead to decreased access to healthcare, which might explain the rise in female mortality and DALYs associated with DMVD. Consequently, future research should concentrate on exploring sex-specific traits in clinical presentations and outcomes among patients who receive suitable interventions, aiming to eradicate sex inequalities in this context.

The impact of DMVD differs among areas characterized by diverse levels of economic and social development. According to the study, all regions experienced an increase in the burden of DMVD, though the extent of this increase varied. Still, overall deaths and DALYs showed a decreasing trend in high and high-middle SDI regions and were lower in low SDI regions than in high SDI regions. This may be related to the comparative adequacy of medical resources and services in developed regions, the generally higher level of education and health insurance coverage of people, and the more developed infrastructure [7]. In further analysis of the relationship between ASIR, ASDR, EAPC, and HDI, during 1990–2019, EAPC of ASDR was significantly negatively correlated with baseline ASDR, while EAPC of ASIR with was positively correlated with baseline ASIR (<45/100,000). For countries with high ASR in 1990, DMVD was more likely to decrease, and possible explanations for this result are: (1) the higher the baseline ASR, the more significant the change in EAPC, (2) countries with high ASR are better able to prioritize DMVD as a high priority for disease prevention programs. In observing the relationship between ASIR, ASDR, EAPC, and HDI, ASIR tended to increase gradually with increasing HDI. In countries with a higher HDI, there was a rise in the ASIR of DMVD. Concurrently, the EAPC of the ASDR was notably lower in regions with high HDI compared to middle and low HDI countries. Moreover, regions with a higher HDI observed a decrease in the ASDR of DMVD. This is consistent with the phenomena we observed in the high and high-middle SDI regions, which, side-by-side, corroborates our study. Regarding DALYs, aging is the most impactful driver in high and high-middle SDI regions. According to the trend of DALYs, it can be observed that epidemiological changes play a driving role in low, low-middle and middle SDI regions, which is an unfavorable phenomenon. Conversely, epidemiological changes negatively affected high and high-middle SDI regions, suggesting they have made some progress in combating DMVD. Studies on disease severity have shown that DMVD is a significantly higher threat to human health in less economically developed regions than in developed regions, which is a severe problem. Consequently, it is imperative for national and regional health departments to proactively explore health and DMVD management strategies that are tailored to their respective economic and health circumstances.

## 5. Conclusions

DMVD remains a significant public health problem that cannot be ignored, despite a decreasing trend in the ASR of global incidence, prevalence, deaths and DALYs from 1990 to 2019. However, we note an adverse development trend in countries with low SDI and seriously aging societies, and sex inequality is particularly prominent. This indicates the need to reposition current prevention and treatment strategies, with some national health administrations developing corresponding strategies for preventing an increase in DMVD based on local health, education, economic conditions, sex differences, and age differences.

## Data Availability

All data were obtained from the open public database: Global Health Data Exchange (GHDx) query tool (http://ghdx.healthdata.org/gbd-results-tool).

## Abbreviations

DMVD, Non-rheumatic degenerative mitral valve disease; GBD2019, Global Burden of Disease, Injury, and Risk Factor Study 2019; UI, uncertainty interval; SDI, Socio-Demographic Index; DALYs, disability-adjusted life years; ASR, age-standardized rate; ASDR, age-standardized DALYs rate; ASIR, age-standardized incidence rate; ASPR, age-standardized prevalence rates; ICD, International Classification of Diseases; EAPC, estimated annual percentage change; CI, confidence interval; BAPC, Bayesian Age-Period-Cohort; HDI, Human development index.

## Supplementary Material

Supplementary material associated with this article can be found, in the online version, at https://doi.org/10.31083/j.rcm2507269.
