# Supplementary material for: Global Epidemiologic Trends and Projections to 2030 in Non-Rheumatic Degenerative Mitral Valve Disease from 1990 to 2019: An Analysis of the Global Burden of Disease Study 2019
Source: Rev Cardiovasc Med. 2024 Jul 22;25(7):269. doi: 10.31083/j.rcm2507269 (PMC11317355; doi:10.31083/j.rcm2507269)
Supplement: Supplementary file 1 [file 2153-8174-25-7-269-s1.docx]

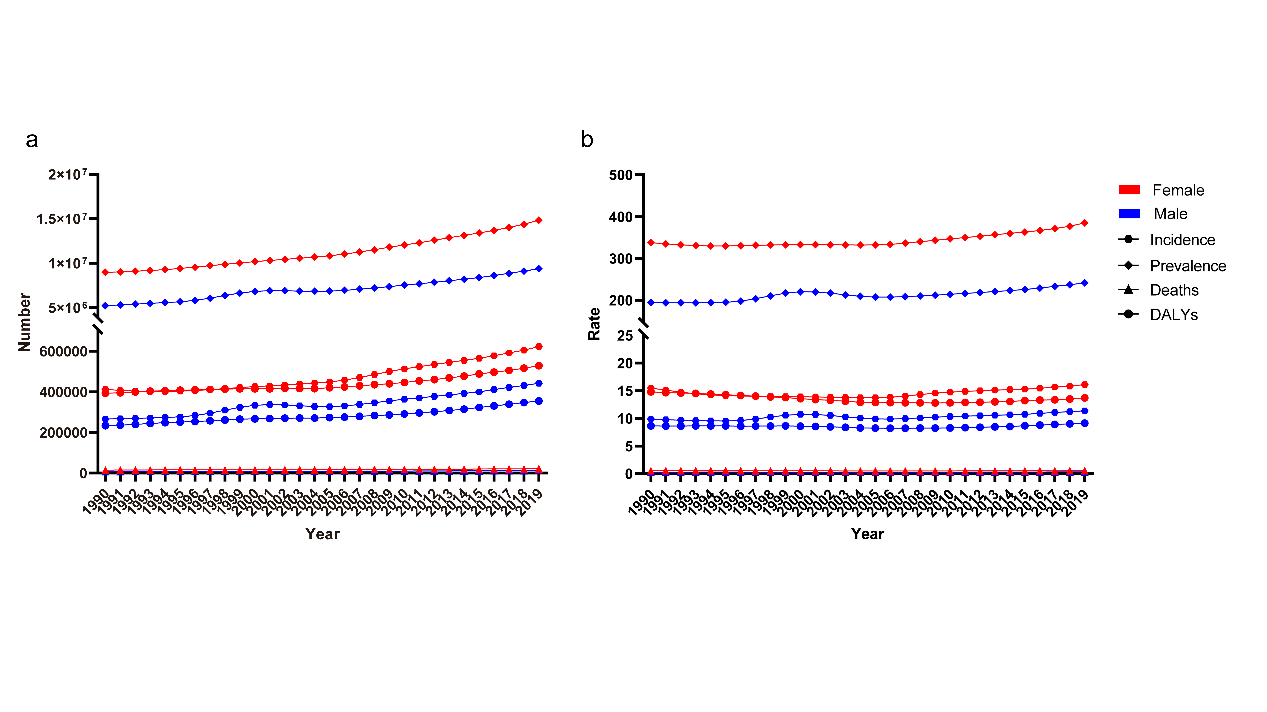


**Supplementary Fig. 1** The number of incidence, prevalence, deaths and disability-adjusted life years, and their rates

for both males and females from 1990-2019


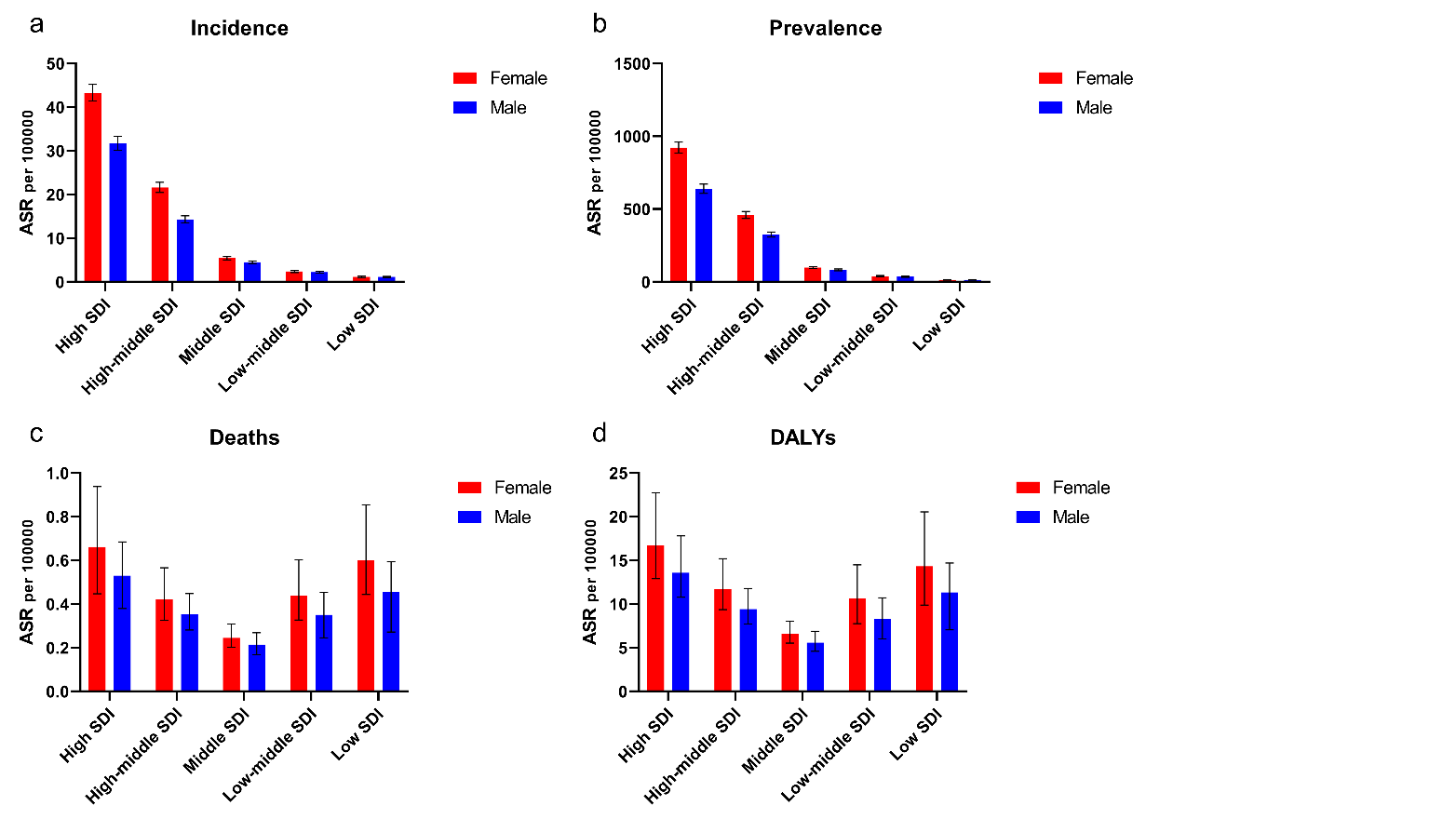


**Supplementary Fig. 2** Differences in Age-Standardized Rate of incidence, prevalence, deaths and disability-adjusted life years between males and females in different SDI regions.


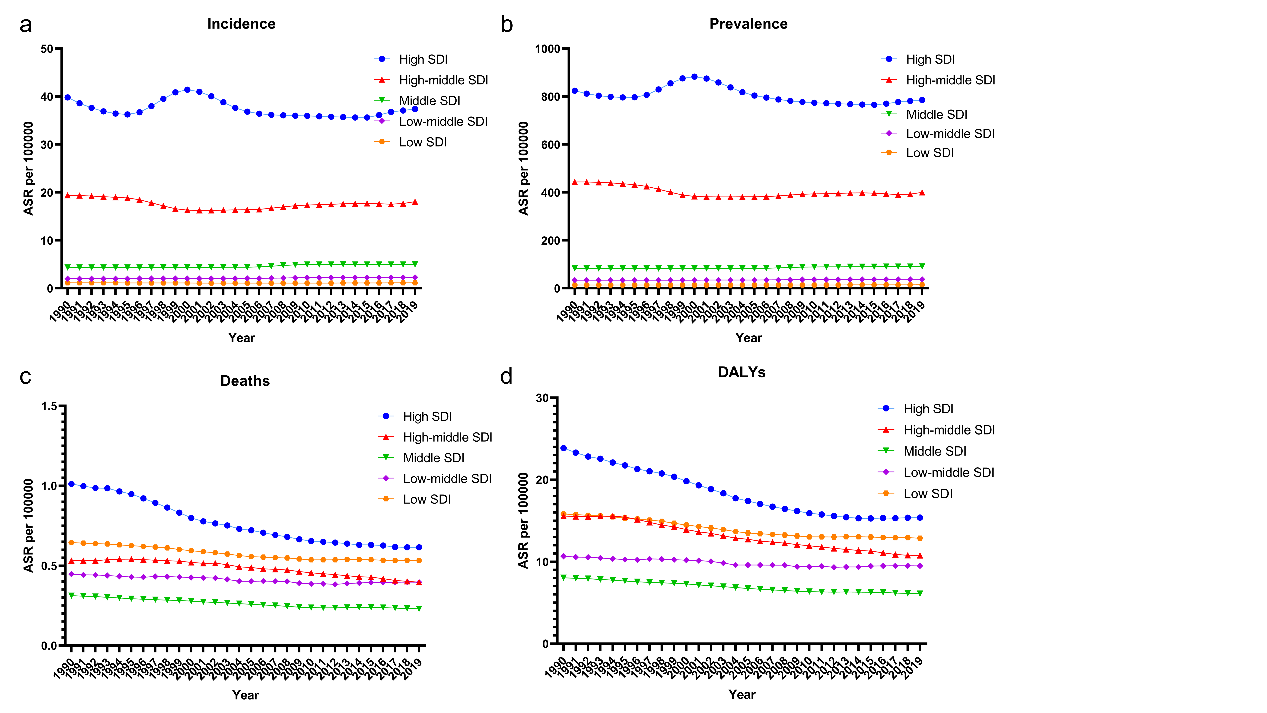


**Supplementary Fig. 3** Epidemiological changes in 5 SDI regions, from 1990 to 2019.

**Supplementary Table 1** BAPC model predicts changes in the incidence of DMVD

| Year | ASR | | | Number | | |
| --- | --- | --- | --- | --- | --- | --- |
|  | Both | Female | Male | Both | Female | Male |
| 1990 | 18.95 | 22.0261 | 15.28814 | 686716.2 | 411333.2 | 275383 |
| 1991 | 18.36 | 21.256 | 14.96894 | 683749.4 | 407359.6 | 276389.8 |
| 1992 | 17.82 | 20.5432 | 14.68127 | 681668.2 | 403911.3 | 277756.9 |
| 1993 | 17.37 | 19.93569 | 14.44464 | 681243.3 | 401636.8 | 279606.5 |
| 1994 | 17.01 | 19.46487 | 14.25168 | 683164 | 401161.8 | 282002.2 |
| 1995 | 16.77 | 19.15847 | 14.10034 | 687859.3 | 403093.4 | 284765.9 |
| 1996 | 16.66 | 18.93105 | 14.12501 | 697633.9 | 406496.9 | 291137 |
| 1997 | 16.7 | 18.74284 | 14.39632 | 713522 | 410692.2 | 302829.8 |
| 1998 | 16.82 | 18.58992 | 14.7939 | 732675.3 | 415346.8 | 317328.5 |
| 1999 | 16.93 | 18.47798 | 15.13551 | 751883.3 | 420671.5 | 331211.8 |
| 2000 | 16.94 | 18.34478 | 15.27506 | 766309.3 | 425485.6 | 340823.7 |
| 2001 | 16.74 | 18.14971 | 15.07463 | 773199.5 | 429469.4 | 343730.1 |
| 2002 | 16.43 | 17.9368 | 14.66263 | 775934.4 | 433691.2 | 342243.2 |
| 2003 | 16.08 | 17.72919 | 14.16794 | 776104.3 | 437764.4 | 338339.9 |
| 2004 | 15.77 | 17.56292 | 13.69538 | 777316.7 | 442704.6 | 334612.1 |
| 2005 | 15.56 | 17.44833 | 13.38426 | 782376.1 | 448379.1 | 333997.0 |
| 2006 | 15.49 | 17.44128 | 13.25171 | 794936.9 | 457254.7 | 337682.2 |
| 2007 | 15.54 | 17.54135 | 13.24221 | 814390.6 | 469924 | 344466.6 |
| 2008 | 15.67 | 17.72502 | 13.31503 | 838252.3 | 485017.7 | 353234.6 |
| 2009 | 15.78 | 17.87663 | 13.38546 | 862453.1 | 500084 | 362369.1 |
| 2010 | 15.83 | 17.94518 | 13.40244 | 883504.7 | 513011.9 | 370492.8 |
| 2011 | 15.76 | 17.87583 | 13.34081 | 901131.7 | 523764.8 | 377366.9 |
| 2012 | 15.68 | 17.7597 | 13.28394 | 919075 | 534296.1 | 384778.9 |
| 2013 | 15.59 | 17.63897 | 13.23067 | 937219.6 | 544868.5 | 392351.1 |
| 2014 | 15.48 | 17.48665 | 13.16285 | 954372 | 554651.5 | 399720.5 |
| 2015 | 15.4 | 17.36629 | 13.11839 | 972272.5 | 564758.6 | 407513.9 |
| 2016 | 15.41 | 17.33395 | 13.16619 | 996016.4 | 577743.5 | 418272.9 |
| 2017 | 15.42 | 17.30123 | 13.21842 | 1020722 | 591179.8 | 429542.6 |
| 2018 | 15.4 | 17.25364 | 13.20971 | 1044024 | 604664 | 439360.1 |
| 2019 | 15.44 | 17.32052 | 13.21365 | 1072331 | 622264.1 | 450066.5 |
| 2020 | 15.1 | 16.86872 | 12.89909 | 1078475 | 625444 | 453031.4 |
| 2021 | 14.99 | 16.75339 | 12.78731 | 1094952 | 635857.5 | 459094.6 |
| 2022 | 14.89 | 16.6447 | 12.67526 | 1111640 | 646565.4 | 465074.6 |
| 2023 | 14.78 | 16.52624 | 12.55949 | 1127991 | 657073.5 | 470917.9 |
| 2024 | 14.66 | 16.38847 | 12.43904 | 1143381 | 666818.2 | 476562.9 |
| 2025 | 14.54 | 16.23578 | 12.31621 | 1157520 | 675656.1 | 481863.7 |
| 2026 | 14.42 | 16.10144 | 12.1994 | 1171942 | 684891.5 | 487050.5 |
| 2027 | 14.32 | 15.9789 | 12.08683 | 1186442 | 694329.7 | 492112.7 |
| 2028 | 14.22 | 15.855 | 11.97535 | 1200847 | 703638.1 | 497208.6 |
| 2029 | 14.12 | 15.72314 | 11.86365 | 1214968 | 712555.8 | 502411.9 |
| 2030 | 14.03 | 15.58855 | 11.75281 | 1228892 | 721142.8 | 507749.6 |

**Supplementary Table 2** Global prevalence, incidence, deaths, and DALYs of DMVD by age group and their rates

| Age | Incidence | | Prevalence | | Deaths | | DALYs | |
| --- | --- | --- | --- | --- | --- | --- | --- | --- |
|  | Number | Rate | Number | Rate | Number | Rate | Number | Rate |
| 15 to 19 | 5706.25 | 0.92 | 9686.92 | 1.56 | 298.32 | 0.05 | 21311.75 | 3.44 |
| 20 to 24 | 17693.46 | 2.95 | 68854.71 | 11.47 | 333.81 | 0.06 | 22244.87 | 3.71 |
| 25 to 29 | 32346.43 | 5.34 | 204613.70 | 33.79 | 357.69 | 0.06 | 22166.34 | 3.66 |
| 30 to 34 | 47159.46 | 7.84 | 414933.30 | 68.96 | 411.24 | 0.07 | 23652.11 | 3.93 |
| 35 to 39 | 59045.24 | 10.91 | 666883.80 | 123.27 | 518.80 | 0.10 | 27605.93 | 5.10 |
| 40 to 44 | 72163.64 | 14.62 | 956486.60 | 193.84 | 706.33 | 0.14 | 34622.64 | 7.02 |
| 45 to 49 | 92737.18 | 19.57 | 1399697.00 | 295.42 | 865.80 | 0.18 | 39501.14 | 8.34 |
| 50 to 54 | 135594.80 | 31.04 | 1835490.00 | 2174.18 | 1291.73 | 0.30 | 53988.94 | 12.36 |
| 55 to 59 | 186621.60 | 50.30 | 1096795.00 | 2522.48 | 1721.17 | 0.46 | 66992.53 | 18.06 |
| 60 to 64 | 179335.00 | 57.38 | 498414.30 | 2956.62 | 2007.84 | 0.64 | 74821.42 | 23.94 |
| 65 to 69 | 134760.10 | 52.11 | 164537.60 | 3447.11 | 2579.16 | 1.00 | 88642.93 | 34.28 |
| 70 to 74 | 72954.44 | 38.99 | 1873466.00 | 428.89 | 3282.05 | 1.75 | 97012.20 | 51.85 |
| 75 to 79 | 20469.04 | 16.11 | 2539210.00 | 684.40 | 4023.89 | 3.17 | 95722.31 | 75.34 |
| 80 to 84 | 4075.93 | 4.83 | 3174601.00 | 1015.76 | 5018.55 | 5.94 | 90757.81 | 107.50 |
| 85 to 89 | 2200.93 | 5.06 | 3546791.00 | 1371.62 | 4976.99 | 11.45 | 68122.30 | 156.67 |
| 90 to 94 | 834.98 | 4.95 | 3249327.00 | 1736.80 | 3743.51 | 22.21 | 38889.66 | 230.70 |
| 95 plus | 216.89 | 4.54 | 2529329.00 | 1990.76 | 2034.37 | 42.62 | 17307.05 | 362.59 |

**Supplementary Table 3** Prevalence, incidence, deaths, and DALYs and their age-standardized rates in female DMVD patients worldwide, between 1990 and 2019

| Year | Incidence | | Prevalence | | Deaths | | DALYs | |
| --- | --- | --- | --- | --- | --- | --- | --- | --- |
|  | Number | ASR | Number | ASR | Number | ASR | Number | ASR |
| 1990 | 411160.70 | 18.16 | 8972285.00 | 419.91 | 14641.69 | 0.76 | 392942.80 | 18.49 |
| 1991 | 407185.80 | 17.58 | 9031744.00 | 412.81 | 14862.93 | 0.75 | 395319.70 | 18.18 |
| 1992 | 403735.70 | 17.07 | 9102459.00 | 406.69 | 15118.92 | 0.74 | 399095.20 | 17.94 |
| 1993 | 401455.20 | 16.63 | 9184879.00 | 401.41 | 15440.29 | 0.74 | 403032.50 | 17.74 |
| 1994 | 400982.70 | 16.30 | 9285211.00 | 396.97 | 15593.05 | 0.73 | 405385.80 | 17.45 |
| 1995 | 402934.30 | 16.07 | 9418352.00 | 394.08 | 15748.06 | 0.72 | 407766.00 | 17.18 |
| 1996 | 406364.60 | 15.90 | 9570534.00 | 391.74 | 15819.81 | 0.71 | 408919.60 | 16.86 |
| 1997 | 410571.30 | 15.77 | 9726803.00 | 389.63 | 15907.52 | 0.69 | 411552.10 | 16.59 |
| 1998 | 415235.80 | 15.66 | 9881079.00 | 387.31 | 15962.53 | 0.68 | 413195.20 | 16.28 |
| 1999 | 420566.60 | 15.56 | 10030668.00 | 384.57 | 16002.73 | 0.66 | 414894.50 | 15.97 |
| 2000 | 425387.20 | 15.44 | 10180347.00 | 381.70 | 15964.56 | 0.64 | 414331.10 | 15.59 |
| 2001 | 429374.60 | 15.25 | 10316405.00 | 377.80 | 16037.70 | 0.63 | 414854.20 | 15.25 |
| 2002 | 433594.40 | 15.06 | 10441771.00 | 373.53 | 16235.88 | 0.62 | 416590.90 | 14.97 |
| 2003 | 437662.80 | 14.86 | 10559533.00 | 368.93 | 16319.12 | 0.61 | 416401.00 | 14.62 |
| 2004 | 442596.30 | 14.68 | 10686218.00 | 364.40 | 16316.28 | 0.60 | 415608.60 | 14.24 |
| 2005 | 448268.20 | 14.53 | 10836237.00 | 360.66 | 16623.76 | 0.59 | 420297.00 | 14.05 |
| 2006 | 457141.10 | 14.47 | 11025233.00 | 357.80 | 16848.49 | 0.58 | 424186.30 | 13.83 |
| 2007 | 469820.00 | 14.54 | 11262993.00 | 356.41 | 17102.56 | 0.57 | 429170.30 | 13.64 |
| 2008 | 484926.00 | 14.66 | 11524597.00 | 355.59 | 17356.82 | 0.56 | 434870.10 | 13.47 |
| 2009 | 499999.10 | 14.77 | 11795982.00 | 354.69 | 17490.90 | 0.55 | 439414.20 | 13.26 |
| 2010 | 512918.00 | 14.78 | 12054275.00 | 353.17 | 17767.15 | 0.54 | 446148.30 | 13.12 |
| 2011 | 523656.50 | 14.72 | 12309412.00 | 350.98 | 18182.59 | 0.54 | 453795.60 | 13.00 |
| 2012 | 534184.80 | 14.66 | 12586057.00 | 349.30 | 18565.34 | 0.53 | 460061.30 | 12.84 |
| 2013 | 544757.10 | 14.59 | 12868565.00 | 347.66 | 18986.20 | 0.53 | 468518.80 | 12.74 |
| 2014 | 554535.20 | 14.51 | 13144325.00 | 345.65 | 19382.65 | 0.52 | 476802.90 | 12.63 |
| 2015 | 564629.20 | 14.42 | 13402245.00 | 343.16 | 19939.51 | 0.52 | 487746.30 | 12.59 |
| 2016 | 577611.40 | 14.42 | 13698493.00 | 341.27 | 20292.81 | 0.51 | 496730.30 | 12.48 |
| 2017 | 591046.50 | 14.43 | 14021170.00 | 339.98 | 20507.86 | 0.50 | 504766.50 | 12.36 |
| 2018 | 604527.90 | 14.42 | 14390605.00 | 339.69 | 20970.98 | 0.50 | 516215.30 | 12.31 |
| 2019 | 622147.60 | 14.51 | 14850959.00 | 341.31 | 21444.41 | 0.49 | 528505.80 | 12.29 |

**Supplementary Table 4** Incidence, prevalence, deaths, and DALYs and their age-standardized rates in male DMVD patients worldwide, between 1990 and 2019

| Year | Incidence | | Prevalence | | Deaths | | DALYs | |
| --- | --- | --- | --- | --- | --- | --- | --- | --- |
|  | Number | Reta | Number | Reta | Number | Reta | Number | Reta |
| 1990 | 266051.80 | 11.98 | 5245818.00 | 279.69 | 7628.41 | 0.52 | 232872.30 | 12.94 |
| 1991 | 267287.20 | 11.75 | 5312090.00 | 276.05 | 7761.80 | 0.51 | 235857.30 | 12.79 |
| 1992 | 268917.60 | 11.55 | 5392039.00 | 273.42 | 7911.95 | 0.51 | 239463.70 | 12.68 |
| 1993 | 271038.00 | 11.39 | 5478235.00 | 271.39 | 8102.49 | 0.51 | 244090.60 | 12.63 |
| 1994 | 273683.80 | 11.26 | 5567910.00 | 269.59 | 8236.95 | 0.51 | 247753.80 | 12.53 |
| 1995 | 276659.50 | 11.16 | 5662056.00 | 268.14 | 8366.95 | 0.50 | 250975.80 | 12.41 |
| 1996 | 283218.20 | 11.20 | 5821300.00 | 269.85 | 8435.56 | 0.49 | 253252.80 | 12.26 |
| 1997 | 295079.80 | 11.44 | 6077961.00 | 276.25 | 8523.01 | 0.49 | 257110.50 | 12.18 |
| 1998 | 309725.40 | 11.78 | 6374495.00 | 284.15 | 8587.09 | 0.48 | 260967.80 | 12.10 |
| 1999 | 323733.60 | 12.07 | 6647530.00 | 290.16 | 8644.35 | 0.47 | 264823.40 | 11.99 |
| 2000 | 333457.60 | 12.19 | 6837630.00 | 291.84 | 8673.26 | 0.46 | 266691.50 | 11.80 |
| 2001 | 336463.10 | 12.03 | 6907968.00 | 287.40 | 8766.17 | 0.45 | 268211.70 | 11.59 |
| 2002 | 335067.30 | 11.71 | 6904651.00 | 279.67 | 8916.75 | 0.45 | 269873.70 | 11.38 |
| 2003 | 331248.30 | 11.32 | 6864145.00 | 270.58 | 9020.93 | 0.44 | 270598.80 | 11.12 |
| 2004 | 327587.30 | 10.94 | 6831056.00 | 261.98 | 9058.43 | 0.43 | 270319.30 | 10.81 |
| 2005 | 327004.90 | 10.69 | 6858575.00 | 256.35 | 9204.68 | 0.43 | 272773.90 | 10.64 |
| 2006 | 330700.60 | 10.57 | 6948282.00 | 253.12 | 9312.82 | 0.42 | 274913.70 | 10.45 |
| 2007 | 337469.40 | 10.56 | 7071877.00 | 251.09 | 9490.35 | 0.42 | 278967.50 | 10.33 |
| 2008 | 346188.10 | 10.61 | 7216393.00 | 249.79 | 9665.20 | 0.41 | 283058.10 | 10.21 |
| 2009 | 355243.30 | 10.66 | 7371636.00 | 248.62 | 9785.77 | 0.40 | 285964.60 | 10.06 |
| 2010 | 363263.80 | 10.67 | 7528955.00 | 247.44 | 9990.60 | 0.40 | 290594.70 | 9.96 |
| 2011 | 370022.00 | 10.63 | 7686804.00 | 245.92 | 10245.88 | 0.40 | 296131.10 | 9.90 |
| 2012 | 377319.70 | 10.60 | 7857886.00 | 244.70 | 10515.36 | 0.40 | 301650.70 | 9.83 |
| 2013 | 384778.60 | 10.57 | 8035911.00 | 243.63 | 10814.19 | 0.40 | 308014.60 | 9.78 |
| 2014 | 392036.30 | 10.54 | 8215516.00 | 242.45 | 11089.96 | 0.39 | 314054.90 | 9.71 |
| 2015 | 399722.60 | 10.51 | 8395364.00 | 241.31 | 11479.19 | 0.40 | 322714.60 | 9.73 |
| 2016 | 410359.00 | 10.56 | 8623369.00 | 241.40 | 11785.29 | 0.39 | 330643.60 | 9.71 |
| 2017 | 421498.60 | 10.63 | 8870472.00 | 241.94 | 12070.63 | 0.39 | 338298.70 | 9.67 |
| 2018 | 431186.60 | 10.65 | 9110004.00 | 242.06 | 12407.16 | 0.39 | 346634.30 | 9.65 |
| 2019 | 441767.80 | 10.69 | 9378157.00 | 242.69 | 12726.86 | 0.39 | 354856.10 | 9.63 |
